# Supplementary material for: Effects of Different Generations and Sex on Physiological, Biochemical, and Growth Parameters of Crossbred Beef Cattle by Myostatin Gene-Edited Luxi Bulls and Simmental Cows
Source: Animals (Basel). 2023 Oct 14;13(20):3216. doi: 10.3390/ani13203216 (PMC10603717; doi:10.3390/ani13203216)
Supplement: Supplementary file 1 [file animals-13-03216-s001.zip › Supplementary Table 1-3, Figure1.pdf]

Table S1 Feed composition

Feed composition	Quality(kg)/day
Silage (kg/head)	12
Gluten (kg/head)	2
Hay (bale/head)	2
Refined feed	2.5

Table S2 Nutrient composition of refined feed

Nutrients	Composition
Crude protein, not less than	16.0
Crude fat, not more than	12.0
Crude fiber, not more than	9.0
Calcium	0.5-1.8
Total phosphorus, not less than	0.4
Sodium chloride	0.8-1.5
Lysine, not less than	0.4

Table S3 The genotyping information of F1 generation

Genotype	Sex	Number of cattle	All
MSTN-g505del(115)	Male	17	44
	Female	27	
MSTN-g507del(6)	Male	13	31
	Female	18	

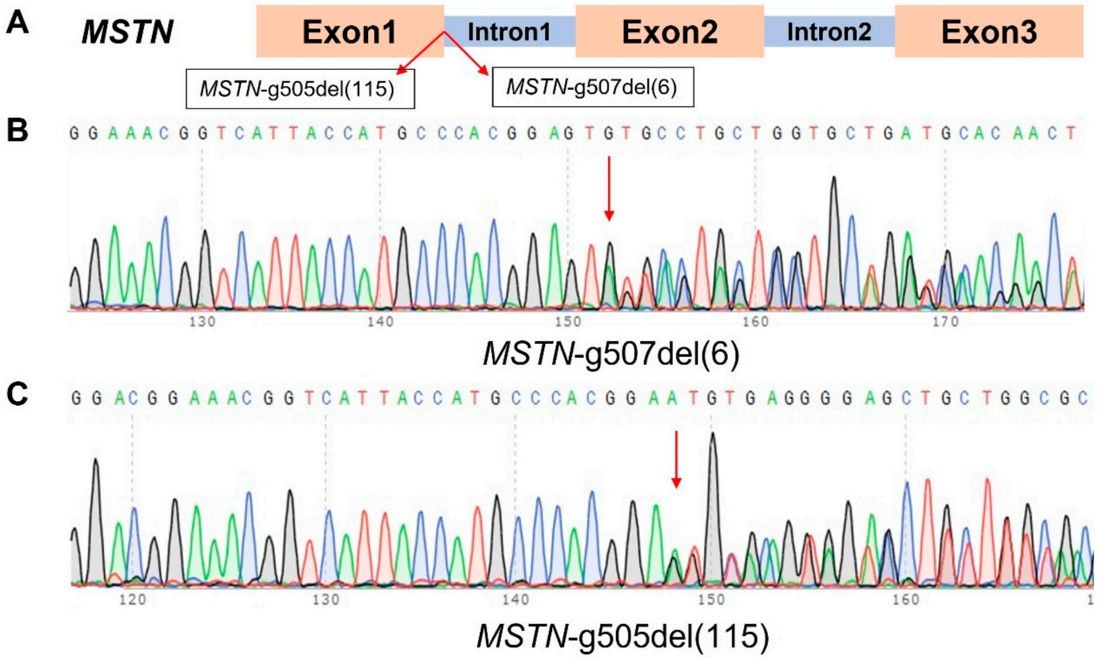

Figure S1. Generation and identification of *MSTN* gene-edited cattle. (**A**) The schematic diagram of *MSTN* gene and the editing sites; (**B**) Sequencing chromas for *MSTN*-g.507del (6) in the *MSTN* gene; (**C**) Sequencing chromas for *MSTN*-g.505del (115)

in the *MSTN* gene.
